# Viral and Latent Reservoir Persistence in HIV-1–Infected Patients on Therapy

**DOI:** 10.1371/journal.pcbi.0020135

**Published:** 2006-10-13

**Authors:** Hwijin Kim, Alan S Perelson

**Affiliations:** Theoretical Biology and Biophysics, Los Alamos National Laboratory, Los Alamos, New Mexico, United States of America; Utrecht University, Netherlands

## Abstract

Despite many years of potent antiretroviral therapy, latently infected cells and low levels of plasma virus have been found to persist in HIV-infected patients. The factors influencing this persistence and their relative contributions have not been fully elucidated and remain controversial. Here, we address these issues by developing and employing a simple, but mechanistic viral dynamics model. The model has two novel features. First, it assumes that latently infected T cells can undergo bystander proliferation without transitioning into active viral production. Second, it assumes that the rate of latent cell activation decreases with time on antiretroviral therapy due to the activation and subsequent loss of latently infected cells specific for common antigens, leaving behind cells that are successively less frequently activated. Using the model, we examined the quantitative contributions of T cell bystander proliferation, latent cell activation, and ongoing viral replication to the stability of the latent reservoir and persisting low-level viremia. Not surprisingly, proliferation of latently infected cells helped maintain the latent reservoir in spite of loss of latent infected cells through activation and death, and affected viral dynamics to an extent that depended on the magnitude of latent cell activation. In the limit of zero latent cell activation, the latent cell pool and viral load became uncoupled. However, as the activation rate increased, the plasma viral load could be maintained without depleting the latent reservoir, even in the absence of viral replication. The influence of ongoing viral replication on the latent reservoir remained insignificant for drug efficacies above the “critical efficacy” irrespective of the activation rate. However, for lower drug efficacies viral replication enabled the stable maintenance of both the latent reservoir and the virus. Our model and analysis methods provide a quantitative and qualitative framework for probing how different viral and host factors contribute to the dynamics of the latent reservoir and the virus, offering new insights into the principal determinants of their persistence.

## Synopsis

Antiretroviral therapy has greatly reduced the mortality of HIV-infected patients. However, even after a decade of suppressive therapy, low levels of virus and latent viral reservoirs persist. Flushing out these reservoirs is a major hurdle that remains to be overcome by anti-HIV therapy. Despite many years of extensive studies, we still lack quantitative understanding of the factors that maintain viral reservoirs and prevent a cure of HIV infection. In this paper, Kim and Perelson develop a novel mathematical model that incorporates the possibility that latently infected cells, like other memory cells, undergo bystander proliferation without being activated. Using the model, they show that T cell bystander proliferation, combined with latent cell activation, enables the stable maintenance of both the latent reservoir and the virus, even in the absence of viral replication. Further, they show that the influence of ongoing viral replication on maintaining the latent reservoir remains relatively insignificant for a range of high drug efficacies. Their results suggest that if the long-term persistence of the latent reservoir results principally from the intrinsic stability of CD4^+^ T cell memory, increasing the potency of anti-HIV therapies may not be sufficient to eradicate HIV.

## Introduction

Quantitative analysis of viral decay characteristics in HIV patients during treatment with antiretroviral therapy (ART) has suggested that the plasma viral load declines in at least three distinct phases (Figure 1). After an initial shoulder period, reflecting both the pharmacokinetic delay of the drugs and the intracellular delay required for a newly infected cell to start producing progeny virus [1,2], the viral load drops exponentially by one to two orders of magnitude during the first two weeks of therapy (the first phase). This reflects rapid viral clearance and the turnover of short-lived productively infected CD4^+^ T lymphocytes with a half-life of less than a day [1,3–5]. Then a slower, second phase of viral decay becomes apparent, with a half-life of 1–4 wk [6], reflecting contributions to plasma virus from a number of sources [6] including populations of longer-lived HIV-infected cells, such as infected macrophages [7], and infected CD4^+^ T cells in a lower state of activation that permit lower levels of viral replication [8], and release of virus from tissue sources such as virus reversibly bound to follicular dendritic cells in the germinal centers of the peripheral lymphoid tissue [9–11]. After several months of ART, plasma HIV-1 RNA in many patients decreases to lower than 50 copies/ml plasma [6,12,13], the limit of detection of current clinical assays. However, even in patients with suppression of viral load of lower than 50 copies/ml for many years, a low level of virus may persist in plasma and other bodily compartments, such as semen, which can be detected by supersensitive assays with a lower limit of detection of 1–5 copies/ml [14–17]. This phase with viral load of lower than 50 copies/ml corresponds to the third phase of viral decay.

**Figure 1:**
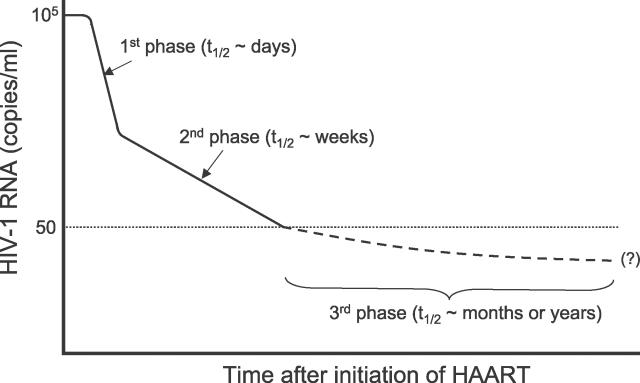
A Schematic Illustration of the Decay Dynamics of HIV-1 after the Initiation of Highly Active ART

Despite extensive studies over the last ten years, the source of this persistent viremia has not been fully elucidated and remains controversial. One possibility is that ART therapy is not fully suppressive and HIV continues to replicate [18], possibly in sites that have poor drug penetration, i.e., in “drug sanctuaries” [19]. Another possibility is that although therapy is fully suppressive, HIV-1 establishes a state of latent infection in resting memory CD4^+^ T cells [20,21], and continued viremia is caused by release of virus due to activation of these latently infected cells [22,23]. Alternatively, both continued replication and release from latent stores contribute.

The decay characteristics of the latent reservoir and plasma viremia in the third phase of viral decay remain subjects of much debate [24]. Initial estimates of the decay half-life of the latent reservoir ranged from 6 mo [25,26] to 44 mo [27,28], which were calculated by assuming a homogeneous latent reservoir exhibiting first-order exponential decay kinetics. However, recent studies by Strain et al. [29,30] have suggested that the pool of latently infected cells contains heterogeneous cell populations that decay at different rates. By measuring the clearance of both HIV-1 DNA and cell-associated infectivity, they estimated that during the first year of treatment the latent reservoir has a median half-life of 18 wk. However, during the subsequent 3 y of treatment, the decay rate declined substantially to a median half-life of 58 wk [30], and moreover, this deceleration appeared to continue.

Studies that focus on the dynamics of plasma HIV-1 RNA in this range of <50 copies/ml are rare. Dornadula et al. [14] reported that in all 22 patients taking suppressive ART they studied, HIV-1 RNA levels were lower than 50 copies/ml (mean level, 17 copies/ml) in the peripheral blood plasma, quantified using the supersensitive RT-PCR assay. Yerly et al. [15] reported the persistence of viremia down to three HIV-1 RNA copies/ml in groups of patients who started ART during acute or chronic infection. Di Mascio et al. [16], using the supersensitive RT-PCR assay, investigated five patients who were selected on the basis of having the greatest degree of viral suppression (no viral blips, no incidents of intercurrent illness, or noncompliance with therapy) among a larger group of more than 80 patients with undetectable viral loads. In three of the five patients, statistically significant viral load decays below 50 copies/ml were observed with a mean half-life of 6 mo, whereas the other two patients did not exhibit any significant decay of plasma viral load. Recently, Coffin and coworkers developed a real-time PCR method to detect and quantify HIV-1 RNA in plasma down to one copy. Using the single-copy assay, they reported that in chronically infected patients, detectable plasma viremia persisted at low levels (in the range of 1–20 copies/ml) even after 7 y of suppressive ART, with a decay half-life of 2 y or even longer (Coffin J (2006) Principles of HIV disease pathogenesis. Presentation at the 13th Conference on Retroviruses and Opportunistic Infections; February 5–8, 2006; Denver, Colorado, United States). The factors influencing the decay kinetics of the latent reservoir and plasma viremia and their relative contributions are still not well-understood.

With an aim toward assessing the broader implications of the maintenance or decay of viral reservoirs, we develop and employ here a simple viral dynamics model describing interactions among viruses, productively infected cells, and latently infected cells. For model parameters and the comparison of model predictions, we adopted data from Strain et al. [30], where they analyzed the decay kinetics of the latent reservoir in 27 patients who initiated treatment during primary infection either before or less than 6 mo after seroconversion. We aim to understand, from a quantitative and integrated perspective, how different viral and host factors may affect the decay characteristic of the latent reservoir and plasma viremia. Our results offer a new perspective into the principal determinants of the stable maintenance of the latent reservoir and the virus.

## Results

### Mathematical Models

A mathematical model describing the production and clearance of HIV-1 and latently infected cells during ART is

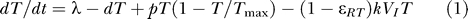


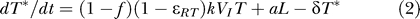


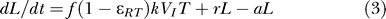


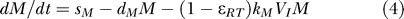


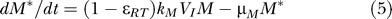


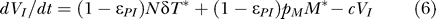


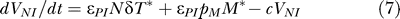
As in previous mathematical models, which describe various aspects of HIV-1 dynamics [1,6,31,32], this model includes *T* representing CD4^+^ T cells that are susceptible to infection (target cells), T* productively infected cells, *L* latently infected cells, *M* long-lived CD4^+^ cells, M* productively infected long-lived cells, *V_I_* infectious virus, and *V_NI_* noninfectious virus-produced by the action of protease inhibitors (PIs), respectively. The total amount of virus *V=V_I_ + V_NI_*. The notion of long-lived infected cells was introduced in [6]. Such cells may include macrophages and monocytes [7], as well as resting CD4^+^ T cells that become infected and produce low levels of virus [8].

Target cells are replenished from their source (thymus) at rate λ, and die at rate *dT*. They are infected by virus, with infectivity *k*, but reverse transcriptase (RT) inhibitors block the viral infection with an efficacy ɛ*_RT_* (0 ≤ ɛ*_RT_* ≤ 1, with ɛ*_RT_* = 1 being a perfect drug), reducing the infectivity by (1 − ɛ*_RT_*). Target cells are also created by proliferation of existing cells, presumably in a density-dependent manner with a slower proliferation as their density gets high. Here this mechanism was represented by a logistic function in which *p* is the maximum proliferation rate and *T_max_* is the target cell density at which proliferation shuts off. Productively infected cells are produced by infection of target cells and by the activation of latently infected cells at rate *aL*. Then they die at rate δT* either from viral cytopathic effects or by host immune response.

The formation of latently infected cells was modeled by assuming a fraction, *f*, of viral infections of target cells results in latency. They may also be created as a result of proliferation of latently infected memory T cells, and die by cell senescence or possibly by a low level of viral cytopathicity or cytotoxic T lymphocyte–mediated killing if they express viral proteins [33,34]. Thus, in this model we are examining the consequences of the assumption that occasional proliferation of (latently infected) memory T cells can occur and that this proliferation does not induce the cell's activation into viral production. In mice cytokines, interferons released during the course of an immune response can induce memory T cells to undergo a single division irrespective of their antigen specificity [35,36]. This type of “bystander” proliferation, which does not activate the cells into clonal expansion, has been hypothesized to be a means by which immunologic memory is maintained. If such proliferation also occurs in human memory CD4^+^ T cells and did not cause the transition from latent to active infection, then it would function to increase the stability of the latent reservoir [37]. This bystander proliferation is not the same as the clonal expansion of latently infected memory T cells that could be caused by interaction with antigen or other stimuli, and hence is not modeled by Equation 1. We assume that any stimulus strong enough to stimulate clonal expansion of latently infected cells, such as appropriate recall antigens, would activate them and thereby cause the transition from latent to active infection. One may explicitly describe the proliferation and death of latently infected cells by defining separate kinetic constants, for example, *p_bs_* for the rate of bystander proliferation and δ*_L_* for the rate of death of latently infected cells. However, here we lumped these into a single parameter, the regeneration rate constant (*r*), by defining *r* = *p_bs_* − δ*_L_*. Then *r* > 0 (*r* < 0) indicates that the net effect of proliferation and death is the generation (clearance) of latently infected cells.

The activation of latently infected cells, which transforms these cells into productively infected cells, functions to decrease the size of the latent reservoir. Here the heterogeneity in the activation rate among latently infected cells [29,30] can be modeled by assigning a non-uniform distribution, instead of a single fixed value, to the activation rate constant (*a*) [38]. Alternatively, one can model the net effect of the heterogeneous cell activation instead of explicitly assigning an arbitrary distribution to *a*. Because cells specific for frequently encountered antigens will be preferentially activated and quickly removed from the latent reservoir, the population of latently infected cells will gradually shift toward cells specific for increasingly rare antigens, resulting in progressive deceleration in the average activation rate of the latent reservoir. We implemented this mechanism by assuming that the activation rate constant exponentially decays in time from its initial value (*a*_0_) to a certain minimum value *a_min_* (0 ≤ *a_min_* ≤ *a*_0_), i.e., *a* = (*a*_0_ − *a_min_*)*e^−^* ^ω*t*^ + *a_min_*, where ω ≥ 0 is the deceleration rate constant.

The remainder of the model is fairly standard [1,6,39]. Long-lived cells are created by a constant source at rate *s_M_*, and die with rate constant *d_M_*. They are infected by virus, with a net infectivity (1 − ɛ*_RT_*)*k_M_*, yielding productively infected long-lived cells, which die at rate μ_M_M*. Virus particles are generated from productively infected cells (T*) and productively infected long-lived cells (M*), where *N* is the number of virions produced during the average lifespan of T* (the burst size), and *p_M_* is the virus production rate by M*. Due to the action of PIs with an efficacy ɛ*_PI_*, however, only a fraction, (1 − ɛ*_PI_*), of the virions are infectious. The sum of infectious and noninfectious virus particles (*V_I_* + *V_NI_*) corresponds to the total plasma viral load (*V*), a quantity typically monitored in a clinical setting. Both types of virus particles are cleared from the plasma with a rate constant *c*.

### Parameter Constraints

Because T cell counts and viral loads in HIV-1–infected patients generally change very slowly before ART is begun, it is reasonable to assume that these and other related system variables are at a quasi-steady state before treatment. This condition then can be used to identify relations existing among parameters and thus to limit the ranges of values parameters can take. At pretreatment, quasi–steady state dT*/*dt* = (1 − *f*)*kVT* + *aL* − δT* = 0 and *dV*/*dt* = *N*δT* + p_M_M* − *cV* = 0. Further, p_M_M_ps_*/*cV_ps_* (= φ_1_), where the subscript *ps* is used to denote a pretreatment quasi–steady state value, correspond to the fraction of plasma virus produced by long-lived infected cells, and *a_ps_L_ps_*/δT_ps_* (= φ_2_) corresponds to the contribution of latently infected cells to the quasi–steady state pool of productively infected cells. Note that φ_2_ can be expanded further, i.e., φ_2_ = *a_ps_L_ps_*/δT_ps_* = (δT_ps_* − (1 − *f*)*kV_ps_T_ps_*)/δT_ps_* = (*N*δT_ps_* − (1 − *f*)*NkT_ps_V_ps_*)/*N*δT_ps_*, to give *N*δT_ps_* = ((1 − *f*)/(1 − φ_2_))*NkT_ps_V_ps_*. Then φ_1_ becomes φ_1_ = p_M_M_ps_*/*cV_ps_* = (*cV_ps_* − *N*δT_ps_*)/*cV_ps_* = (*cV_ps_* − ((1 − *f*)/(1 − φ_2_))*NkT_ps_V_ps_*)/*cV_ps_* = (*c* − ((1 − *f*)/(1 − φ_2_))*NkT_ps_*)/*c*, yielding the following parameter constraint:

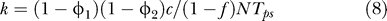
where φ_1_ and φ_2_ have been estimated to be 0.01–0.07 and <0.01, respectively [6], implying that *k* < *c*/*NT_ps_*.

### Model Reduction

By applying several clinically valid assumptions and relations among the above variables and parameters, the proposed model can be reformulated as follows: first, after a transient increase during the initial 4–8 wk of treatment, overall CD4^+^ T cell counts in patients on suppressive therapy increase slowly [3,40,41]. Further, the proportion of productively infected cells in the peripheral blood CD4^+^ T cell population in patients on therapy is generally small (≤0.05%) [21], with higher frequencies found in gut-associated lymphoid tissue [42–44]. Therefore, as a first approximation, it may be reasonable to assume that the concentration of target cells (*T*) remains constant. Later, we will examine the case in which *T* increases. If *T* = constant = *T*_0_, where *T*_0_ denotes a quasi–steady state value of *T* that can differ from one patient to the next depending on the parameters characteristic of the virus and host, then Equation 1 can be ignored in the analysis. Second, because of the limited contribution of long-lived infected cells to plasma viral loads (1%–7% in patients before treatment) [6,8], their contributions to new rounds of viral infection and virus production in patients on therapy will be ignored in the main text. In the Materials and Methods section, we will detail the effects of including this population in our analysis and also show by simulation that for viral loads lower than 50 copies/ml long-lived infected cells still make a small contribution to total virus. Thus, Equations 4 and 5 and the terms containing p_M_M* in the Equations 6 and 7 will be ignored in the subsequent analysis. Third, because *V* = *V_I_* + *V_NI_*, the summation of Equations 6 and 7 leads to the equation *dV*/*dt* = *N*δT* − *cV*. Note that this equation can also be obtained by substituting *V_I_* = (1 − ɛ*_PI_*)*V* and *V_NI_* = ɛ*_PI_V* into Equations 6 and 7, respectively. Before therapy, ɛ*_PI_* = 0, *V* = *V_I_*, and *V_NI_* = 0, and thus the conditions *V_I_* = (1 − ɛ*_PI_*)*V* and *V_NI_* = ɛ*_PI_V*, are satisfied. Once therapy is begun (ɛ*_PI_* ≠ 0), although these conditions are not satisfied initially, simulations show that with typical parameters, after a few days the ratio of infectious and noninfectious viruses approaches *V_I_*/*V_NI_* = (1 − ɛ*_PI_*)/ɛ*_PI_*. Therefore, the condition *V_I_* = (1 − ɛ*_PI_*)*V* should be satisfied at the beginning of the third phase of viral decay, and it can be substituted into the above differential equations to express the system in terms of a measurable quantity *V*. Finally, one can define a composite parameter ɛ as (1 − ɛ) = (1 − ɛ*_RT_*)(1 − ɛ*_PI_*), and then ɛ represents the total combined drug efficacy [31,45]. Below we analyze the resulting differential equations:

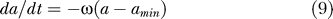


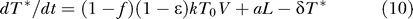


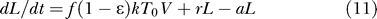


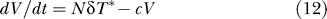



### Model Parameters

For the baseline CD4^+^ T cell count before treatment (*T_ps_*), we adopted the value, *T_ps_* = 486 cells/μl, from Strain et al. [30], which was the median value for all 27 patients who initiated treatment during primary infection either before (13 out of 27 patients) or less than 6 mo after seroconversion (14 out of 27 patients). While most primary infection patients are identified after the viral peak, it is uncertain whether all attained their viral set point. Nonetheless, this CD4^+^ T cell count is a reasonable pretreatment quasi–steady state value. We assume that the third phase of viral decay (with viral load below 50 copies/ml) began 3 mo after initiation of ART [29], which corresponds to the initial time point of the proposed model. By this time point, most viral replication should be suppressed so that the latent pool should not be rapidly being replenished any longer, allowing for the possibility of its depletion. The CD4^+^ T cell count at this time point (*T*_0_) was calculated by assuming that CD4^+^ T cells increased by 109 cells/μl during the first 3 mo of ART [46], i.e., *T*_0_ = 595/μl. The concentration of latently infected cells at this time point was calculated from Strain et al. [30], where the median value for all the patients was slightly less than one infectious unit per million cells (≤0.9 infectious unit per million). Because the total number of CD4^+^ T cells may be close to 1.2 × 10^11^ cells after the first 3 mo of ART [47], we assumed that the total number of latently infected cells with replication-competent viral genomes at this time point (*L*_0_) was 10^5^ cells. In the case of the virus, assuming HIV distributes equally throughout the body's extracellular water, and that the typical 70-kg individual has 15 l of extracellular water, then *V*_0_ = 50 copies of HIV-1 RNA/ml × 15,000 ml = 7.5 × 10^5^ HIV-1 RNA copies. Other initial conditions of the proposed model, such as *T*_0_ *** and *a*_0_, were chosen to match clinically observed decay characteristics of the latent reservoir and the virus, which will be discussed later.

The death rate of productively infected cells (δ) and the virion clearance rate (*c*) were set to our current best estimates, δ = 1 d^−1^ [5] and *c* = 23 d^−1^ [48], respectively. The in vivo burst size (*N*) is not well-known. Estimates based on counting the number of HIV-1 RNA molecules in an infected cell vary between 100 and a few thousands [49,50], while estimates based on viral production have been as high as 5 × 10^4^ (Chen HY, Di Mascio M, Perelson A, Gettie A, Ho D, et al. Determination of virus burst size in vivo using a single-cycle SIV in rhesus macaques. Presentation at the 9th Conference on Retroviruses and Opportunistic Infections; February 24–28, 2002; Seattle, Washington, United States). In our analysis, we chose *N* = 2 × 10^4^ HIV-1 RNA/cell as a baseline value. For a standard three drug–therapy regime containing one PI and two RT inhibitors, the total drug efficacy (ɛ) was estimated to range from 0.68 to 0.75 [51], and ɛ = 0.7 was applied as its baseline value. The fraction of new viral infections resulting in latency (*f*) is not well-known, but based on previous work [31] we chose *f* = 3 × 10^−6^ as a baseline value such that there exist approximately ten latently infected cells per million CD4^+^ T cells at the pretreatment quasi–steady state [30]. The viral infectivity (*k*) was calculated using one of the above pretreatment steady state conditions. Specifically, *k* = (1 − φ_1_)*c*/(*NT_ps_*) (obtained by assuming *f* ≈ 0 and φ_2_ ≈ 0 in Equation 8) was used as a default since we felt the contribution of φ_1_ (= 0.01 − 0.07) to the value of *k* should not be ignored. For the given parameter values, *k* = 2.248 × 10^−6^ μl (RNA copy)^−1^ d^−1^ when φ_1_ = 0.05. Although it has been estimated that memory/effector cells (CD45RO^+^) have a half-life of 1–2 mo (or equivalently a decay rate of 0.012 − 0.023 d^−1^) [52], this rate represents the summation of their death and activation rates, which are not necessarily the same as the corresponding rates (δ*_L_* and *a*) for latently infected cells. Further, because several factors, including the reversion of activated CD4^+^ T cells to resting memory CD4^+^ T cells and the bystander proliferation of memory CD4^+^ T cells (*p_bs_*), contribute to the generation of memory CD4^+^ T cells, even if a steady state is assumed for the overall memory CD4^+^ T cell count [41], individual parameter values for *p_bs_* δ*_L_*, and *a* cannot be estimated. Therefore, we estimated a lumped parameter *r* (= *p_bs_* − δ*_L_*) and ω, one of the parameters for *a*, by nonlinear least-square fitting of the proposed model to relevant data, as will be explained below. Finally, unless otherwise specified, the effects of viral evolution, which may continuously change parameter values, and the possible cell density–dependency of some parameters including δ were ignored [31,53].

### Steady State Analyses

Steady states (or equilibrium points) of the proposed system (Equations 9–12) can easily be found. In Materials and Methods we examine the stability of the uninfected steady state, i.e., [*ā*, 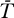
*, 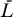, 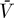] = [*a_min_*, 0, 0, 0], where overbars denote steady state quantities, via eigenvalue analysis. In the special case that the minimum activation rate of the latent reservoir is zero (*a_min_* = 0), the eigenvalues have the relatively simple form (see Materials and Methods):

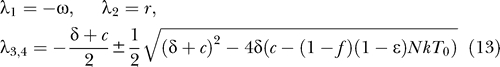
In the special case of 100% effective therapy (ɛ = 1), the eigenvalues of the system are


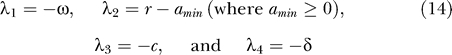


For the uninfected steady state to be (locally) stable, all the eigenvalues should be negative. When *a_min_* = 0, this requires that ω > 0 and *r* < 0 (i.e., the latently infected cell bystander proliferation rate (*p_bs_*) is less than the death rate (δ*_L_*)). Because all the parameters are positive and *f*, ɛ ≤ 1, (δ + *c*)^2^ − 4δ(*c* − (1 − *f*)(1 − ɛ)*NkT*_0_) ≥ (δ + *c*)^2^ − 4δ*c* = (δ − *c*)^2^ ≥ 0, indicating that both λ_3_ and λ_4_ are real, and that the eigenvalue with the negative square root term (λ_3_) is negative. Lastly, λ_4_ < 0 is satisfied if *c* > (1 − *f*)(1 − ɛ)*NkT*_0_, or equivalently ɛ > ɛ*_c_*, where the critical efficacy [31,32] is


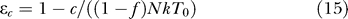


Because the reproductive number (*R*) for this system, which represents the average number of secondary infected cells produced by a single infected cell [54], corresponds to *R* = (1 − *f*)(1 − ɛ)*NkT*_0_/*c*, this condition can also be expressed as *R* < 1. Because *a* monotonically approaches *a_min_* (≥ 0) as *t* increases, the system approaches a linear system as *t* increases, implying that no asymptotically stable nontrivial periodic orbit exists, and thus the satisfaction of these stability conditions also guarantees the global (asymptotic) stability of the steady state. Therefore, the last condition implies that given *a_min_* = 0, ω > 0 and *r* < 0, if clearance of the virus in its host is fast enough, or therapy is effective enough, the latent reservoir and the virus are predicted eventually to be cleared from its host. That this is not observed suggests that we should focus on the condition under which the uninfected state is unstable.

The stability of the uninfected steady state is changed, or equivalently a bifurcation occurs, when any of the eigenvalues (or their real parts when they are complex numbers) becomes zero. Typically a manifold of equilibria other than the steady state at the origin exists at this point, thereby allowing for the possibility of the stable maintenance of the latent reservoir and the virus at finite positive values. For example, when the magnitude of the minimum activation rate of the latent reservoir is zero (*a_min_* = 0), from Equation 13 an eigenvalue becomes zero if ω = 0, *r* = 0, or *c* = (1 − *f*)(1 − ɛ)*NkT*_0_ (or equivalently *R* = 1). Because *a* approaches zero as *t* increases (Equation 9), the steady state condition of Equations 10–12 becomes (1 − *f*)(1 − ɛ)*kT*_0_ *V* − δT* = 0, *f*(1 − ɛ)*kT*_0_ *V* + *rL* = 0, and *N*δT* − *cV* = 0, respectively. If *c* = (1 − *f*)(1 − ɛ)*NkT*_0_, the first and third equations above become identical, implying that steady states with positive values of productively infected cells and plasma virus (T* > 0 and *V* > 0) satisfying the relation *N*δT* − *cV* = 0 exist. The second equation, *L* = *f*(1 − ɛ)*kT*_0_ *V*/(−*r*) implies that for a positive steady state value of *V*, the level of latently infected cells (*L*) has a finite positive value if *r* < 0, i.e., their death rate (δ*_L_*) is greater than the bystander proliferation rate (*p_bs_*). If this were not the case, then latently infected cells would continuously expand.

In general, when *a_min_* ≥ 0 (for 0 ≤ ɛ ≤ 1), the bifurcation condition of the system becomes (see Materials and Methods for a detailed derivation):

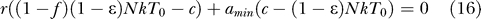
This can be used to derive a more general form of the critical efficacy, i.e.,

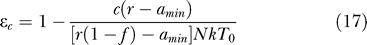
However, since *f* ≪ 1, this critical efficacy essentially reduces to ɛ*_c_* = 1 − *c*/(*NkT*_0_), equivalent to Equation 15 when *f* ≪ 1. If we use the pretreatment steady state condition *NkT_ps_* = (1 − φ_1_)*c*, the critical efficacy becomes ɛ*_c_* = 1 − *T_ps_*(*r − a_min_*)/((1 − φ_1_)[*r*(1 − *f*) − *a_min_*]*T*_0_) ~ 1 − *T_ps_*/((1 − φ_1_)*T*_0_). This suggests the critical efficacy depends on patient-dependent parameters (i.e., the fraction of viral production by long-lived cells (φ_1_), and the ratio of the pretreatment CD4^+^ T cell count (*T_ps_*) to the CD4^+^ T cell count at the beginning of the third phase of viral decay (*T*_0_)). Note that in the calculations above, *T*_0_ is also the T cell count at the uninfected steady state. Because in this initial calculation we have assumed the T cell count does not vary, *T*_0_ is in fact an underestimate of the T cell count that would be attained if virus were eliminated and T cell recovery occurred. Thus, the critical efficacy calculated above is lower than previous estimates [31,32], which replace *T*_0_ by the T cell count of a healthy individual.

Once parameters pass through the bifurcation condition, the uninfected steady state becomes unstable. The implications of this instability will be discussed later.

These steady state analyses give the insights into long-term behavior of the proposed system, and also provide necessary conditions to maintain low, but nonzero steady state levels of the latent reservoir and the virus under drug therapy. However, they cannot provide much insight into dynamic characteristics of the system, including decay profiles of the latent reservoir and the virus, and the time required to reach a steady state. In the following sections, we shall examine dynamic behavior of the system.

### Decay Kinetics Data of Latent Reservoir and Estimation of Model Parameters

We first consider data on the decay kinetics of the latent reservoir. Strain et al. [30] studied HIV-1–infected patients who initiated treatment during primary infection. They estimated that the latent reservoir has a median decay half-life of 18 wk during the first year of treatment (weeks 4–48), followed by a slower decay with a median half-life of 58 wk during the subsequent three years of treatment. To make progress with our analysis, we assumed that this group of patients exhibited the greatest degree of decay of the latent reservoir, based on this decay half-life, compared with other studies with a half-life of 6–44 mo [25–28], and the time interval after initiation of ART to reach viral load below 50 copies/ml (4–12 wk [29,30]), compared with 3–6 mo in other studies [26,55]. We call this the maximal decay profile of the latent reservoir. Although ART might not completely stop ongoing virus replication in this case, for simplicity we assumed ɛ ≈ 1, implying that the decay profile of the latent reservoir was determined entirely by the relative contributions of the effects of cell activation (*a*) and cell regeneration (*r*). Therefore we employed Equations 9–12 with ɛ = 1 as the system model to explain the above data. The effect of ongoing virus replication (ɛ < 1) will be discussed later.

For dynamic simulations of the model, the initial conditions (at 3 mo after initiation of ART) were chosen as described in Materials and Methods. The parameters *a_min_*, ω, and *r* also need to be specified. For simplicity, we assume here that *a_min_* = 0. The effect of *a_min_* > 0 (or the persistence of low-level activation of the latent reservoir) will be discussed later. The remaining parameters, ω and *r*, were estimated by nonlinear least-square fitting of the model (specifically ln(*L*)) to the latent reservoir decay (specifically ln(*L^data^*)). Here the decay kinetics data (*L^data^*) were generated by using the median decay slopes of the latent reservoir for all 27 patients in Strain et al. [30], under the assumption that the third phase of viral decay began at 3 mo (13 wk) after initiation of ART and that each phase of latent reservoir decay (weeks 13–48 and subsequent three years) exhibited exponential decay kinetics. For example, we applied the following formula to mimic the Strain data: for 0 ≤ *t* ≤ *t*_1_, *L^data^* = *L*_0_exp(*k_d_*_1_ *t*), and for *t* > *t*_1_, *L^data^* = *L*_0_exp(*k_d_*_1_ *t*_1_)exp(*k_d_*_2_(*t* − *t*_1_)), where *t*_1_ = (48–13) wk, *k_d_*_1_ = −0.038 /week (t_1/2_ ≈ 18 weeks), and *k_d_*_2_ = −0.012 /week (t_1/2_ ≈ 58 weeks). The data (open circles) and the best-fit curve generated from the model are shown in Figure 2A; the corresponding parameter estimates are *r* = −0.00171 (± 0.00007, 95% CI) d^−1^ and ω = 0.00939 (± 0.00064, 95% CI) d^−1^. If *a_min_* > 0, *a* approaches *a_min_* (Equation 9) and thus *dL*/*dt* approaches *dL*/*dt* = (*r* − *a_min_*)*L* (Equation 11) as *t* increases. Therefore, the estimate of *r* in this case may be closer to −0.00171 + *a_min_*, or equivalently *r* − *a_min_* ≈ −0.00171 d^−1^. These are consistent with the result from the steady state analysis that indicated that in order for the level of the latent reservoir to approach zero, *r* < *a_min_* should be satisfied. If ɛ < 1, the estimate of *r* will be less than −0.00171 + *a_min_* due to the contribution from ongoing viral replication (*f*(1 − ɛ)*kT*_0_ *V*). To examine this, we set ɛ = 0.7, kept ω fixed at its estimated value, and found that with *a_min_* = 0, the best-fit value of *r* = −0.00205 d^−1^. Further, the increased viral replication, which can also be achieved by increasing *a_min_*, enhanced the fit of the theoretical curve to the data during the early decay phase (unpublished data).

**Figure 2:**
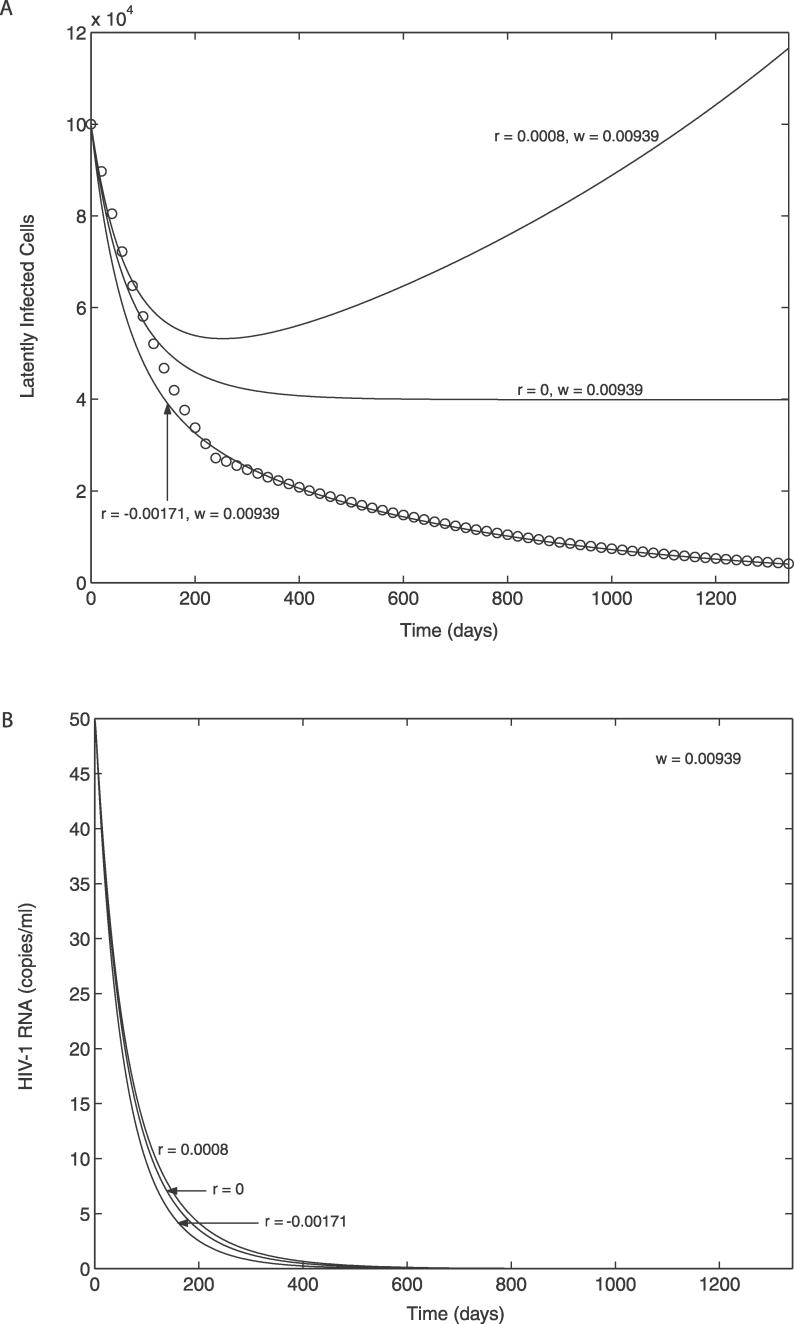
Effect of the Bystander Proliferation of the Latent Reservoir on the Latent Reservoir and on Plasma Viral Load When there is no ongoing viral replication (ɛ = 1) and the minimum activation rate of the latent reservoir is zero (*a*_min_ = 0). The open circles indicate the decay kinetics of the latent reservoir suggested by Strain et al. [30], where they found t_1/2_ ≈ 18 wk up to week 35 and t_1/2_ ≈ 58 wk for the subsequent 3 y. The solid curve with *r* = −0.00171 d^−1^ and ω = 0.00939 d^−1^ represents the best-fit curve to the data. (A) Latent reservoir. (B) Plasma viral load.

### Decay Characteristics of Latent Reservoir and Virus without Ongoing Viral Replication

We next investigated how the decay profiles of the latent reservoir and plasma virus may be affected by variations in parameters. We specially focused on examining the possibility of maintaining plasma virus at a low, but nonzero steady state level solely by activation of latently infected cells (without any contribution from ongoing viral replication, i.e., ɛ = 1) without severely depleting the latent reservoir. The steady state analysis (Equation 14) suggests that the regeneration rate of latently infected cells (*r*) is a unique parameter that can change the stability of the uninfected steady state, and that its stability changes at *r* = *a_min_* when ω > 0, allowing for the possibility of the stable maintenance of the latent reservoir and the virus at nonzero steady states. To check the possibility, we computed the time profiles of both the latent reservoir and viral load when *r* = *a_min_* = 0 and ω was maintained at its estimated value (ω = 0.00939 d^−1^). As shown in Figure 2B, the latent reservoir approaches and stays at a nonzero steady state. However, the viral load approaches zero (Figure 2B). The same was true even when we increased *r* beyond its bifurcation value (*r* = *a_min_* = 0). For example, when *r* = 0.0008 d^−1^, the level of the latent reservoir blew up (Figure 2A), while viral load kept approaching zero (Figure 2B). These can be explained as follows: because ω > 0 and *a_min_* = 0, *a* approaches zero as *t* increases (Equation 9). Hence dT*/*dt* becomes dT*/*dt* = −δT* as *t* increases (Equation 10), and thus T* and the production of virus approaches zero as *t* → ∞, leading to *V* → 0 as *t* → ∞. However, under this condition *dL*/*dt* becomes *dL*/*dt* = *rL* as *t* increases (Equation 11). Therefore, the latent reservoir can either reach a nonzero steady state level (*r* = 0) or blow up (*r* > 0), while the virus is always eliminated. Note that although we assumed a constant positive *r* value (*r* = 0.0008 d^−1^) for the case with *r* > 0, the regeneration rate of latently infected cells should decrease as CD4^+^ T cell counts increase, causing the driving force for bystander proliferation to diminish. Therefore, the level of the latent reservoir would be expected to reach a stable steady state instead of blowing up as observed in Figure 2A.

These results suggest that if the magnitude of the minimum activation rate of the latent reservoir is (or is close to) zero (*a_min_* ≈ 0), the stable maintenance of viral load at a nonzero steady state level cannot be achieved without ongoing viral replication. However, several genetic analyses have demonstrated that in patients under ART with viral loads below 50 copies/ml, at least some portion of the viruses have genetic sequences similar to those found in integrated provirus in resting memory CD4^+^ T cells which were infected prior to ART [22,56–58]. This suggests that these plasma viruses originated from the latent reservoir and that activation of latently infected cells may be occurring in these patients (*a_min_* > 0).

### Effect of Persistence of Low-Level Activation of the Latent Reservoir (when ɛ = 1)

As a next step, we examined whether the persistence of low-level activation of the latent reservoir (*a_min_* > 0) would enable maintenance of both the latent reservoir and plasma virus at nonzero steady state levels without any contribution from ongoing viral replication. To check the possibility, we numerically solved the model, Equations 9–12, for *a_min_* = 0, *a*_0_/3, and *a*_0_/2, with ω fixed at its previously estimated value (ω = 0.00939 d^−1^), and for *a_min_* = *a*_0_ (an upper extreme, when ω = 0), at the bifurcation condition *r* = *a_min_* (where the stability of the uninfected steady state is changed). Figure 3 shows that if *a_min_* is large enough, both the latent reservoir and plasma virus can reach nonzero steady states with non-negligible magnitudes within the time period of interest. Thus, if low-level activation of the latent reservoir persists with a non-negligible magnitude, the viral load can be stably maintained at a finite positive value without severely depleting the latent reservoir, and without any contribution from ongoing viral replication, as long as *r* (= *p_bs_* − δ*_L_*) ≈ *a_min_* > 0. Therefore, *a_min_* itself can be a major factor influencing the persistence of the latent reservoir and the virus.

**Figure 3:**
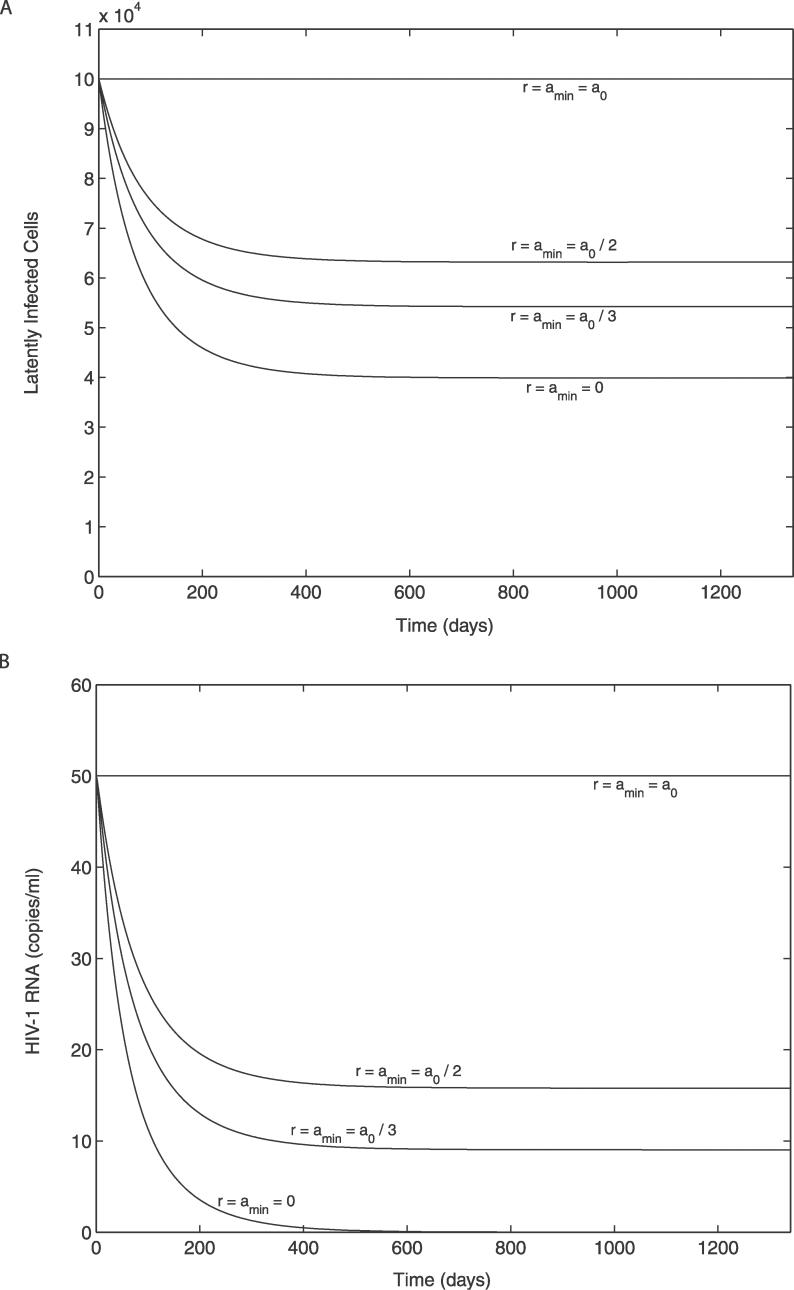
Effect of Persistent Low-Level Activation of the Latent Reservoir on the Latent Reservoir and on Plasma Viral Load When there is no ongoing viral replication (ɛ = 1) and the rate of bystander proliferation of the latent reservoir is equal to the minimum activation rate (*r* = *a_min_*), i.e., at the bifurcation condition. (A) Latent reservoir. (B) Plasma viral load.

### Effect of Ongoing Viral Replication on the Decay Characteristics of the Latent Reservoir and the Virus

We next investigated how variations in the drug efficacy (ɛ) and the resulting variations in the extent of ongoing viral replication affect the decay dynamics of the latent reservoir and plasma virus. For simplicity, we consider the case of zero minimum activation of the latent reservoir (*a_min_* = 0), where the stability of the uninfected steady state is changed at ɛ = ɛ*_c_* = 1 − *c*/((1 − *f*)*NkT*_0_). Using the given parameter values (*T_ps_* = 486/μl, φ_1_ = 0.05, *f* = 3 × 10^−6^, and *T*_0_ = 595/μl), ɛ*_c_* = 0.1402. As noted above, because we are assuming *T* is constant, this value of ɛ*_c_* is artificially low. The critical efficacy is increased if *T*_0_ is increased, or if *T* rather than being constant increases with time on therapy [46,59]. Clearly, if more target cells are available, it becomes more difficult to slow or halt viral replication.

We computed the time profiles of both the latent reservoir and plasma virus for ɛ values above, at, and slightly below the critical efficacy, while ω and *r* were maintained at their previously estimated values, i.e., *r* = −0.00171 d^−1^ and ω = 0.00939 d^−1^. As expected, for ɛ > ɛ*_c_*, both the latent reservoir and the virus decay toward zero (Figure 4A and 4B). When ɛ = ɛ*_c_*, the viral load stays at a nonzero steady state (Figure 4B). However, although the latent cell population will ultimately reach a nonzero steady state value, *L* = *f*(1 − ɛ)*kT*_0_ *V*/(−*r*), since *r* = −0.00171 < 0; nevertheless, *L* kept decreasing for at least ten years (unpublished data), consistent with the decay observed by Siliciano et al. [28,60]. When ɛ was reduced to slightly below the critical efficacy, the viral load kept increasing (Figure 4B). Although the latent reservoir initially decayed, it ultimately increased as the viral load increased and finally blew up. These blow-ups occur because we have assumed the target cell level is constant, thus there are always more cells to infect. When target cells are limited, this behavior is prohibited. For example, if the equation *dT*/*dt* = λ − *dT* + *pT*(1 − *T*/*T_max_*) − (1 − ɛ)*kVT* (= *f(T*,*V*)), obtained by substituting *V_I_* = (1 − ɛ*_PI_*)*V* into Equation 1, is added to the current model, then, as studied elsewhere [31,32], instead of blowing-up, an infected steady state is reached in which 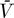 = λ/((1 − ɛ)*k* 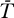) + (*p*(1 − 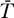/*T_max_*) − *d*)/((1 − ɛ)*k*).

**Figure 4:**
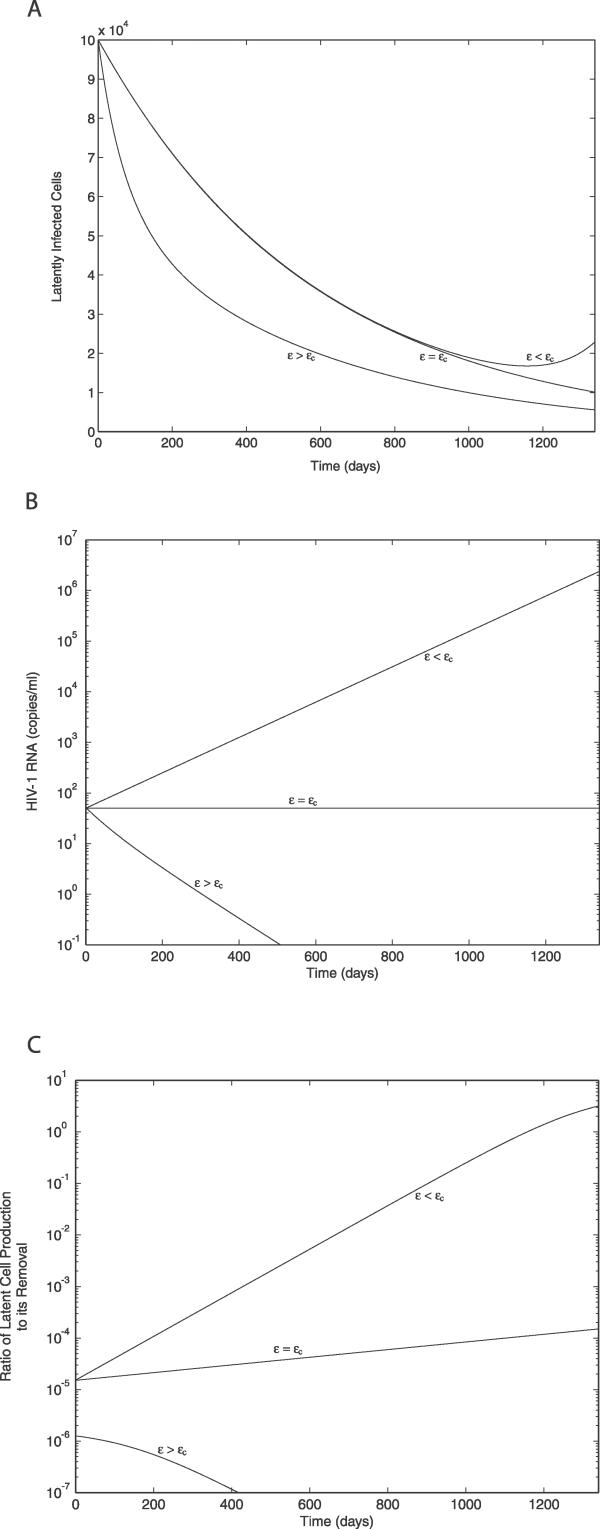
Effect of Ongoing Viral Replication (ɛ < 1) on the Latent Reservoir, Plasma Viral Load, and the Contribution of Ongoing Viral Replication to the Level of the Latent Reservoir Measured by the Ratio of the Rate of Production of Latently Infected Cells by Ongoing Viral Replication to the Net Removal Rate of Latently Infected Cells The results were obtained for ɛ values above (ɛ = 0.7 > ɛ*_c_*), at (ɛ = 0.1402 = ɛ*_c_*), and slightly below (ɛ = 0.133 < ɛ*_c_*) the critical drug efficacy (ɛ*_c_*) when *a_min_* = 0, *r* = −0.00171 d^−1^, and ω = 0.00939 d^−1^. (A) Latent reservoir. (B) Plasma viral load. (C) Contribution of ongoing viral replication.

To better understand the contribution of ongoing viral replication to the level of the latent reservoir, we computed *Ra* = |*f*(1 − ɛ)*kT*_0_ *V*/(*r* − *a*)*L*|, i.e., the ratio between the rate of production of latently infected cells by ongoing viral replication and the net removal rate of latently infected cells (since *r* = −0.00171 < 0 and *a* ≥ 0). Here, *Ra* = 1 indicates that the generation of latently infected cells equals its clearance, and thereby leads to a steady state of the latent reservoir if *Ra* is maintained at 1 as *t* increases. As shown in Figure 4C, when ɛ *>* ɛ*_c_*, i.e., ɛ = 0.7, the rate of virus-induced production of latently infected cells remained less than 0.0002% of its net clearance rate (or *Ra* < 2 × 10^−6^) throughout the entire time course, leading to a rapid decay of the latent reservoir (Figure 4A). When ɛ = ɛ*_c_*, *Ra* increased the level the latent reservoir (*L*) decreased, and the viral load (*V*) was constant. Nevertheless, *Ra* remained below 1, even after 10 y of ART (unpublished data), implying that a steady state of the latent reservoir was not reached within this time period. It was the same even when a higher fraction of viral infection resulting in latency (*f*) was assumed, for example, its potential maximum value *f* = 0.05 [61] (and ɛ*_c_* = 0.095). When ɛ < ɛ*_c_*, i.e., ɛ = 0.133, *Ra* increased more rapidly and passed through 1 as the viral load increased.

These results suggest that for ɛ ≥ ɛ*_c_* the contribution of ongoing viral replication to the level of the latent reservoir remains insignificant. However, under the condition (*r* − *a_min_* < 0), where the level of the latent reservoir would approach zero if there were not any ongoing viral replication, such as for the group of patients considered here with *r* − *a_min_* ≈ −0.00171 d^−1^, ongoing viral replication may function to maintain both the latent reservoir and the virus at nonzero steady state levels. Further, ɛ < ɛ*_c_* may serve as a necessary condition for their persistence, although the extent of ongoing viral replication at ɛ*_c_* itself may not be enough to achieve the stable maintenance of the latent reservoir. Finally, we also found that as the fraction of infection events that lead to latency (*f)* increases, at the corresponding critical efficacy, the time to reach the steady state of the latent reservoir is reduced, increasing the possibility of its stable maintenance (unpublished data).

Although these studies could be extended further to include parameter sensitivity analyses, as studied elsewhere [62,63], given our intended focus and the limited space, we chose not to elaborate on this point.

### Effect of Variations in ω

We also examined the role that variations in ω play. Because the eigenvalues of the system except λ_1_ = −ω do not depend on ω, when ω > 0, any variation in ω does not affect the steady states of the system and its stability, but it simply affects the kinetics of the system. For example, an increase in ω generally increases the level of the latent reservoir before its steady state is reached by reducing its loss caused by activation. Conversely, the viral load is transiently reduced due to a reduced generation of productively infected cells, suggesting that any variation in ω transiently drives the level of the latent reservoir and the viral load in opposite directions before their steady states are reached.

### Effect of T Cell Recovery on Decay Characteristics of Latent Reservoir and Virus

During ART, CD4^+^ T cells increase but with a rate of increase that tends to slow as the duration of therapy increases [46,59]. Using the equation *dT*/*dt* = λ − *dT* + *pT*(1 − *T*/*T_max_*) − (1 − ɛ)*kVT*, a modified form of Equation 1, the dynamics of T cell recovery can be modeled. While this equation, and forms with *p* = 0, have been commonly written, it is not at all clear that this is a good model for CD4^+^ T cell recovery under ART. Independent of the value of *p*, this model typically yields a strong predator–prey type oscillation between virus (*V*) and T cells (*T*) instead of the monotonic increase in the T cell concentration observed in most HIV patients under suppressive therapy. Further, model parameters, such as λ and *d*, have been estimated for healthy individuals, and thus they may not be reliable estimates to describe the T cell recovery in HIV patients, especially for patients with significantly impaired thymic functions. Thus, we decided to follow an approach introduced by Perelson et al. [1] where the empirical data on CD4^+^ T cell recovery was used rather than the modified Equation 1. Thus, we modeled the recovery of T cells and its progressive deceleration by assuming the following saturation kinetics:

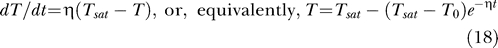
where η (≥0) is a rate constant characterizing the extent of deceleration of T cell recovery, and *T_sat_* is the T cell concentration that *T* approaches as *t* → ∞. Here a theoretical maximum value of *T_sat_* is the CD4^+^ T cell count observed in HIV-negative individuals (≈800–1,200 cells/μl [64–66]). Adding Equation 18 to Equations 9–12 with *T*_0_ replaced by *T* in Equations 10 and 11 gives an extended system with the following uninfected steady state: [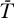, *ā*, 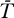
*, 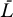, 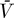] = [*T_sat_*, *a_min_*, 0, 0, 0]. The Jacobian matrix evaluated at this steady state is

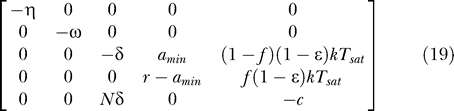
Two of the eigenvalues of this Jacobian are −η and −ω. The other eigenvalues are given by Equation 20 in Materials and Methods, with *T*_0_ replaced by *T_sat_*. This suggests that all the results obtained previously should remain qualitatively the same except that a bifurcation now occurs at a condition based on the value of *T_sat_* instead of *T*_0_. For example, when *a_min_* = 0, the critical efficacy of this system is ɛ*_c_* = 1 − *T_ps_*/((1 − φ_1_)(1 − *f*)*T_sat_*). Because *T_sat_* ≥ *T*_0_, for given values of *T_ps_*, φ_1_, and *f*, this critical efficacy is larger than the one based on *T*_0_, implying that the range of drug efficacies required to clear the latent reservoir and the virus is narrower than under the assumption that the concentration of T cells remained constant during the third phase of viral decay. Figure 5 shows how the critical efficacy depends on *T_sat_*. The dotted line shows one estimate of the drug efficacy for a standard three drug–therapy regime (1 PI + 2 RT inhibitors), i.e., ɛ ≈ 0.7 [51], and the dashdot line shows the critical efficacy at *T*_0_. Clearly, an increase in the T cell concentration may function to provide more target cells for viral infection, increasing the extent of ongoing viral replication and thereby requiring a higher drug efficacy to clear the virus (and also the latent reservoir). The result also suggests that a slow but continued increase in CD4^+^ T cell counts and the resulting recovery of immune functions can ironically increase a risk of rapid amplification of viral replication and thereby accelerate disease progression unless therapy remains effective enough during the entire time course. It is quite similar to the case of opportunistic infection that transiently activates the immune system, enhancing viral replication and thereby replenishing the latent reservoir unless therapy is 100% effective [53].

**Figure 5:**
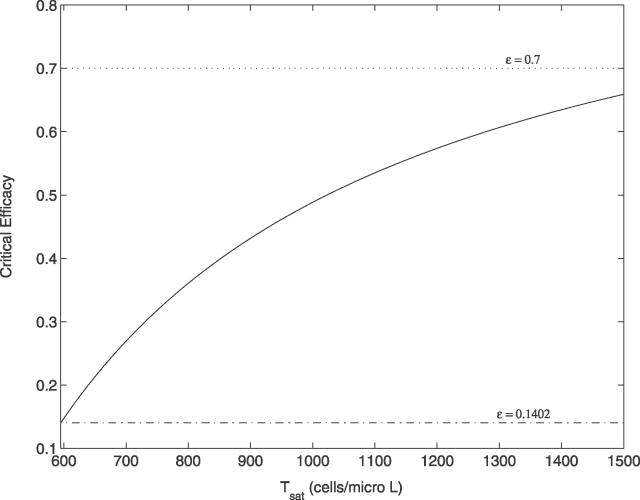
Dependency of the Critical Drug Efficacy on the Concentration of T cells *T_sat_* represents the concentration that T cells approach as the duration of ART increases. The solid line shows how the critical efficacy depends on *T_sat_*. The dotted line indicates a typical drug efficacy estimated for standard combination ART (ɛ ≈ 0.7), and the dashdot line indicates the critical efficacy when *T_sat_* = *T*_0_ = 595 cells/μl, i.e., ɛ*_c_* = 0.1402.

Finally, despite an increase in the critical efficacy as *T* increases with time on therapy, the critical efficacies for a clinically relevant range of *T_sat_* remained below an estimate of the drug efficacy for a standard three drug–therapy regime. Then why is the elimination of the virus not observed in patients on standard ART? This apparent contradiction may be explained as follows. First, the estimate of the efficacy of standard ART may not be accurate. Louie et al. [51] compared the relative efficacy of standard ART with a highly potent four-drug regime and found standard ART to be 68% as effective as this new regime. Clearly, the four-drug regime may not be 100% effective. Moreover, drug efficacies are not constant in time. Dixit et al. [67] by taking into account the pharmacokinetics of the PI ritonavir estimated that the efficacy of this drug could vary between 0.4 and 0.85 between dosing intervals, further complicating estimating the efficacy of combination ART. Also, in the range of viral loads of interest in this study (<50 copies/ml), the drug efficacy for standard therapy may be influenced by the effects of drug sanctuaries [19]. If most residual replication is occurring in such sanctuaries, as has been postulated in some models [19], then drug efficacy in the sanctuary could be significantly lower than the estimate of 0.7. Hence the actual drug efficacy may be much closer to, if not below, the critical efficacies than what was observed in Figure 5. Second, the model studied in the main text has been simplified and long-lived infected cells ignored. If such cells are included in the model then, as we show in Materials and Methods, the critical efficacy is increased. Intuitively, this makes sense as drug therapy now also needs to block infection in this additional compartment. Third, although we found that for most *r* values satisfying *r* < *a_min_* (≥0), the critical efficacy is relatively insensitive to variations in *r* or *a_min_*, it rapidly approaches 1 as *r* closely approaches *a_min_* (see Equation 17). Therefore, if the *r* values in many HIV-1 patients are closer to their *a_min_* values than in the group of patients considered here (i.e., *r* − *a_min_* ≈ −0.00171 d^−1^), the corresponding critical efficacies may be higher than the drug efficacy of standard therapy. Last, even if standard therapy has ɛ > ɛ*_c_*, the time for viral elimination could be very long under the current standard therapy, making it appear as if the virus will persist.

## Discussion

We have developed a new viral dynamic model that incorporates the idea that latently infected CD4^+^ T cells that have specificities for common antigens will be activated more frequently than latently infected cells with specificities for rare antigens. We incorporated this effect by assuming that *a*, the rate of latent cell activation, decreases with time on therapy until it reaches a minimum level, *a_min_*. We also took into consideration the possibility that latently infected cells could proliferate without becoming activated into virus production. In Figure 6A we show that under ART this model gives rise to a rapid first-phase viral decay, followed by a slower “second-phase” decay. Here, however, the second phase rather than being strictly exponential (i.e., linear on a log plot) has some curvature. For assays with a lower cutoff of 400–100 copies/ml, as was typical at the time two-phase decay was defined [6], this second phase would be close to exponential. However, our model suggests that with assays that can detect viral load decays to much lower limits, e.g., five copies/ml, curvature should be seen in the viral decay profile. However, one might speculate that at low plasma viral loads, i.e., <50 copies/ml, residual viral replication is occurring mostly in “drug sanctuaries” [68]. If this were the case, then the average drug efficacy, ɛ, might be lower than it was initially, slowing the viral decay at long times. Whether curvature and possible slowing of viral decay curves occurs at long times remains to be examined experimentally.

**Figure 6:**
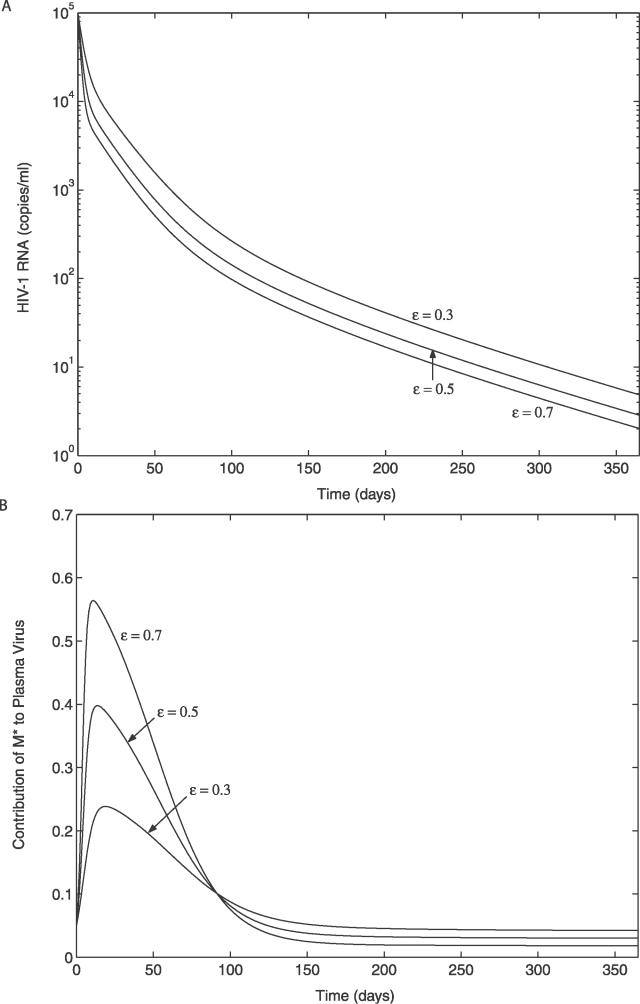
Simulated Decay Dynamics of HIV-1 after the Initiation of ART The initial conditions and parameters used in the simulation are given in Materials and Methods. The results are shown for three different drug efficacies (ɛ = 0.3, 0.5, and 0.7). (A) Viral decay profile. (B) Contribution of long-lived infected cells to plasma virus (φ_1_(*t*) = p_M_M*(*t*) / (*N*δT*(*t*) + p_M_M*(*t*))).

From the model one can also predict the contribution of long-lived infected cells to plasma virus. Figure 6B shows that for the parameters we use, long-lived infected cells make a substantial contribution to plasma virus only during the first few months of therapy, but by the time the viral load decays below 50 copies/ml their contribution is relatively inconsequential. Thus, when focusing on events that occur when viral loads are below 50 copies/ml, i.e., the third phase of viral load decay, we simplified our model by ignoring long-lived infected cells. How virus and latently infected cells persist during this third phase was the main focus of our modeling.

Siliciano and colleagues have hypothesized that the long-term persistence of the HIV-1 latent reservoir may result principally from its intrinsic stability in patients with HIV-1 RNA <50 copies/ml [37]. Our analysis suggests that if latently infected cells can undergo occasional bystander proliferation without transitioning into active viral production, then there are circumstances under which this hypothesis may be true. For example, if the net rate of proliferation of latently infected cells is greater than or equal to their minimum rate of activation, i.e., *r* > *a_min_*, then the latent reservoir can be autonomously maintained. Further, if *a_min_* *>* 0, then continuous activation of latently infected cells will provide a source of plasma virus even when drug efficacies are sufficiently high (ɛ ≥ ɛ*_c_*) to block most ongoing viral replication. Thus, our modeling shows that there are circumstances under which the longevity of the latent reservoir can be maintained without ongoing virus replication that serves to continually replenish the latent reservoir.

Viral replication is inevitably accompanied by sequence evolution, including the selection of drug-resistant mutants. Therefore, if the stability of the latent reservoir is maintained primarily by ongoing viral replication (under the condition that the latent reservoir cannot maintain its stability autonomously, i.e., *r* < *a_min_*), viral sequences from both the latent reservoir and plasma virus should rapidly be replaced by replicating (or recently evolved) plasma virus that may be genetically distinct from wild-type or archival proviral genomes generated prior to the initiation of ART. The replicating plasma virus may also have a greater genetic distance from the inferred most recent common ancestor, with possibly a higher fitness in the context of current therapy, compared with wild-type or archival viruses originated from the latent reservoir. However, wild-type or archival viruses have been found to persist in blood plasma of aviremic patients under ART [22,56–58], arguing against the possibility that the latent reservoir is stably maintained solely by ongoing viral replication without any mechanism to improve its intrinsic stability, such as bystander proliferation.

Recent experimental studies provide more direct evidence supporting the idea that the latent reservoir stability may result principally from the intrinsic stability of long-lived memory CD4^+^ T cells. For example, Strain et al. [29] used the M184V drug resistance mutation in HIV RT as a genetic marker to identify and track subpopulations of the latent reservoir replenished by ongoing viral replication during lamivudine-containing antiviral therapy. They found that cells infected before treatment initiation, containing the wild-type HIV-1 DNA, exhibited a much slower decay than those infected during treatment, defined by the M184V marker. Thus, the long-lived nature of part of the latent pool may reflect more the stability of immunologic memory, possibly maintained by bystander proliferation, rather than its replenishment by ongoing viral replication. A study by Monie et al. [60,69] further supports this idea of the intrinsic stability of the latent reservoir. Instead of aviremic patients, they examined viremic patients who have high levels of viral replication and immune activation, presumably with a higher turnover of the latent reservoir than aviremic patients. They compared HIV-1 *pol* sequences from stably integrated, replication-competent viral genomes in the latent reservoir with actively replicating drug-resistant viruses in the plasma of patients failing ART. They found that the latent reservoir dominantly harbored wild-type HIV-1 and archival drug-resistant viral species despite a fitness disadvantage of these viruses compared with the contemporaneous plasma viruses, consistent with the findings by Strain et al. [29].

Taken together, the intrinsic stability of latently infected resting memory CD4^+^ T cells may be of primary importance in determining their long-term persistence. Although ongoing viral replication may also affect the stability of the latent reservoir, the contribution of ongoing viral replication seems to be relatively insignificant. Further, ongoing viral replication alone, independent of the extent of its influence on the latent reservoir dynamics, cannot simultaneously explain the observed long-term persistence and genetic composition of the latent reservoir, highlighting the role of nonviral mechanism in maintaining latent reservoir stability. This suggests that in many HIV-1 patients the net regeneration rate of the latent reservoir (*r*) is maintained comparable to its minimum activation rate (*a_min_*), or much closer to *a_min_* than in the group of patients considered here (*r* − *a_min_* ≈ −0.00171 d^−1^). This suggestion, of course, needs to be tested experimentally.

### Therapeutic Implications

If the dynamics of the latent reservoir is dominated by its intrinsic stability, while the contribution of ongoing viral replication to the reservoir dynamics is relatively insignificant, this may have significant implications for the design of anti-HIV therapies. To eradicate HIV-1 from patients, it would then be essential to flush out the latent reservoir during suppressive antiviral therapy, such as by the activation of resting memory CD4^+^ T cells [70,71] or the selective expression of HIV-1 from latently infected cells by an agent such as valproic acid [72]. Otherwise, the intrinsic stability of the latent reservoir may guarantee lifetime persistence of HIV-1 since replication-competent HIV-1 recovered from the latent reservoir is sufficient to rapidly rekindle infection once therapy is stopped [73]. Further, the above results argue against an immune modulation strategy intended to reduce cellular activation and thereby minimize the release of virions from the latent reservoir [74]. This strategy could be useful only under the hypothesis that the continuous replenishment of the latent reservoir by ongoing virus replication is the principal determinant of the reservoir stability [75]. Early treatment initiation, such as during primary infection, also seems to be beneficial in reducing the population size of the latent reservoir since it is established early in infection [30] and then may persist autonomously. However, drug toxicity, cost, and development of resistant mutants, caused by prolonged treatment periods, are also important factors to be considered.

Treatments with greater potency (treatment intensification) [76,77] will also be required to better control viral replication, and thereby minimize its contribution to the stability of the latent reservoir since ongoing viral replication may still contribute to replenishment of the latent reservoir in aviremic patients on current ART regimens [26,76] and even to somewhat higher extents in some cases [29]. It has been reported that in some chronically infected patients intensification of standard ART accelerates the decay of both plasma viremia and the latent reservoir, as well as decreases the frequency of intermittent viremia (blips) [76,77]. More important, better suppression of viral replication may be a critical prerequisite for the success of activation-based strategies, since otherwise the activation of resting memory CD4^+^ T cells may function to boost viral replication by providing more target cells for viral infection and thereby could even increase the population size of the latent reservoir. Further, in this case, the population of latently infected cells will gradually shift toward cells harboring the contemporaneous plasma viruses that may have a higher fitness in the context of current therapy compared with wild-type or archival viruses dominant in the latent reservoir population before activation. In parallel with developing treatments with greater potency, efforts to address the effects of host factors, such as cellular drug efflux proteins, differential drug metabolism, and drug sanctuary sites, will be needed since these factors limit antiviral drug efficacy. In spite of potential benefits, treatment intensification (or host factor control) alone will not be able to eradicate HIV-1 if the latent reservoir is self-sustaining.

### Conclusions

By developing and employing a simple viral dynamic model, we studied how different viral and host factors may affect the decay characteristics of the latent reservoir and plasma virus in HIV-infected patients who initiated treatment during primary infection. We focused on identifying key factors that enable the stable maintenance of the latent reservoir and plasma virus at low, but nonzero steady state levels. We identified three major factors: 1) bystander proliferation of latently infected cells, characterized by the net proliferation rate *r,* 2) the magnitude of persistent activation of the latent reservoir, characterized by *a_min_*, and 3) the extent of ongoing viral replication, controlled by the drug efficacy ɛ. We also identified necessary conditions for the persistence of both the virus and the latent reservoir. We found that the bystander proliferation rate of latently infected cells, *r*, dominates the dynamics of the latent reservoir. Further, how much effect variations in *r* have on the dynamics of free virus depended on the magnitude of the minimum activation rate of latently infected cells (*a_min_*). For example, as *a_min_* approached zero, any variation in *r* had little quantitative or qualitative effect on the dynamics of the virus, and the persistence of the virus could not be achieved merely by increasing *r* when there was little (ɛ > ɛ*_c_*) or no ongoing viral replication. As *a_min_* increased, the effect of latent cell proliferation on the dynamics of the virus became stronger. Thus, when a certain level of activation of the latent reservoir was maintained during the entire time course, the viral load could be stably maintained at a low steady state without severely depleting the latent reservoir, even without any viral replication. These results suggest that latent cell activation can be a major factor influencing the persistence of the latent reservoir and the virus. We also found not surprisingly that the extent of ongoing viral replication (or ɛ) dominates the dynamics of the virus. Further, when bystander proliferation was not significant, i.e., *r* < *a_min_*, for drug efficacies at or above the critical efficacy (ɛ ≥ ɛ*_c_*), the influence of ongoing viral replication on the dynamics of the latent reservoir remained insignificant irrespective of the value of *a_min_*, and thus the stable maintenance of the latent reservoir could not be achieved. However, a decrease in ɛ (increasing viral replication) below its critical efficacy (ɛ*_c_*) can lead to a stable infected steady state with nonzero levels of the latent reservoir and the virus.

Finally, we investigated how progressive recovery of T cells would affect the dynamics of the latent reservoir and the virus. We found that all the above results remain qualitatively valid except that bifurcation conditions depend on the concentration that T cells approach as *t* → ∞ (*T_sat_*) instead of the constant T cell concentration (*T*_0_). This result suggests that the range of drug efficacies required to clear the latent reservoir and the virus may be much higher than the model predictions based on the assumption of a constant T cell concentration, and may contribute to the failure of current therapies.

Taken together, our results suggest that the persistence of the latent reservoir and the virus, observed in the vast majority of HIV patients on suppressive ART, may result from a combined contribution of intrinsic physiological parameters of patients, such as *r* and *a_min_*, that characterize the behavior of latently infected cells, and ongoing viral replication—the extent of which may also be affected by patient-dependent drug efficacy and rates of T cell recovery. The work also clearly raises the issue of the importance of testing experimentally the fundamental idea of the paper, i.e., that latently infected cells are memory cells that may undergo bystander proliferation. Such proliferation does not generate clonal expansion of memory cells, and we suggest that it might not trigger latently infected cells into active viral production. If this is the case, then the stability of the latent reservoir is much easier to understand and our model provides quantitative testable predictions about the behavior of this reservoir and plasma viremia below the limits of detection of standard clinical assays.

## Materials and Methods

### Eigenvalues of the system.

The Jacobian matrix evaluated at the uninfected steady state ([*ā*, 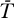
*, 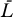, 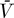] = [*a_min_*, 0, 0, 0]) of the proposed system (Equations 9–12) is

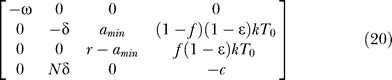
The characteristic equation computed from this Jacobian has one factor of (λ_1_ + ω), indicating that one of the eigenvalues is λ_1_ = −ω. The other eigenvalues are the solutions of the characteristic equation of the following submatrix:

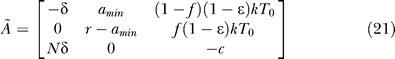
Here the characteristic equation is

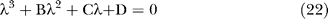
where

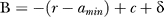


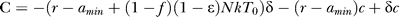


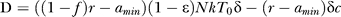



The analytical expressions for the eigenvalues are too complicated to be presented here. However, in special cases such as *a_min_* = 0 or ɛ = 1, the eigenvalues have much simpler forms, as given in Equations 13 and 14, i.e.,

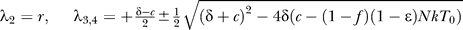
 when *a_min_* = 0,

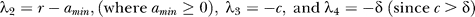
when ɛ = 1.

### Generalized bifurcation condition of the system.

If *a_min_* > 0 (for ω ≥ 0), the eigenvalues are too complicated to easily find bifurcation conditions directly from them (unless ɛ = 1). Alternatively, bifurcation conditions can be obtained by finding conditions to make the linear system *ÃX* = 0 singular, where *Ã* is from Equation 21 and *X* = [T*, *L*, *V*]*^T^*, implying that there exist nontrivial solutions of *X* as well as the trivial solution *X* = 0. For example, the linear system can be reduced to the following equivalent system by Gaussian elimination:

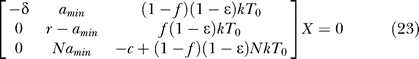
If *r* = *a_min_*, then only ɛ = 1 can make the system singular, leading to an infinity of solutions of *X*. If *r* ≠ *a_min_*, then one more step of elementary row operation yields *r*((1 − *f*)(1 − ɛ)*NkT*_0_ − *c*) + *a_min_*(*c* − (1 − ɛ)*NkT*_0_) as the pivot in this elimination step (or the (3, 3) diagonal element of the reduced matrix). For this reduced system to be singular, this term should be equal to zero, yielding the generalized bifurcation condition as given in Equation 16, i.e.,

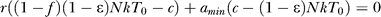
Note that if ɛ = 1, *r* should be equal to *a_min_* (since c > 0) to satisfy the singular condition, and thus the bifurcation condition remains valid for all the ɛ values. Further, when *a_min_* = 0, the bifurcation condition is simplified to *r* = 0 or *c* = (1 − *f*)(1 − ɛ)*NkT*_0_, and ω = 0 simply represents *a_min_* = *a*_0_ (= *a*), indicating that the bifurcation condition is valid for all the *a_min_* and ω values.

### Initial conditions.

Initial conditions for productively infected cells (T*) and the activation rate of latently infected cells (*a*) were chosen by the following procedure. We assumed that in these patients with the maximal latent reservoir decay, the latent reservoir steadily decays, at least on average, from the beginning of the third phase of viral decay (*t* = 0 in the model). The same assumption was applied for viral load, based on the study by Di Mascio et al. [16], where in three of the five patients having the greatest degree of viral suppression with undetectable viral loads, statistically significant viral load decays below 50 copies/ml were observed with a mean half-life of 6 mo. Here we neglected the effects of transient episodes of viremia, i.e., viral blips. For the system model to explain the steady decay of viral load, *dV*/*dt* = *N*δT* − *cV* ≤ 0, implying that *T*_0_ *** ≤ *cV*_0_ */N*δ. To avoid an abrupt decay of virus at *t* = 0, we chose the maximum value of *T*_0_ ***, i.e., *T*_0_ *** = *cV*_0_ */N*δ. To determine *a*_0_ = *a*(0), we note that dT*/*dt* = (1 − *f*)(1 − ɛ)*kT*_0_ *V* + *aL* − δT* ≤ 0 should be satisfied for viral load to decay steadily in the third phase, implying that *a*_0_ ≤ δ*T*_0_ ***/*L*_0_ − (1 − *f*)(1 − ɛ)*kT*_0_ *V*_0_/*L*_0_ = *cV*_0_ */NL*_0_ − (1 − *f*)(1 − ɛ)*kT*_0_ *V*_0_/*L*_0_. We chose the maximum value, i.e., *a*_0_ = *cV*_0_ */NL*_0_ − (1 − *f*)(1 − ɛ)*kT*_0_ *V*_0_/*L*_0_ (for example, *a*_0_ = 0.0058 d^−1^ when ɛ = 0.7), in order to avoid an abrupt decay of T* (and thus *V*) at *t* = 0. Finally, for the steady decay of the latent reservoir, *dL*/*dt* = *f*(1 − ɛ)*kT*_0_ *V* + *rL* − *aL* ≤ 0, implying *r* ≤ *a*_0_ − *f*(1 − ɛ)*kT*_0_ *V*_0_/*L*_0_, providing an upper bound of *r*.

### Contribution of long-lived cells to bifurcation condition of the system.

The system model (Equations 9–12) can be extended to include the contribution of long-lived cells as follows:

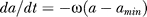


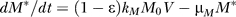


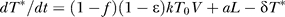


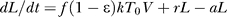


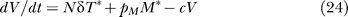
This system has the following uninfected steady state: [*ā*, 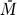
*, 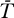
*, 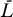, 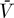] = [*a_min_*, 0, 0, 0, 0], and the Jacobian matrix evaluated at this steady state is

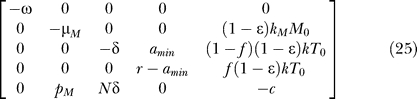
One of the eigenvalues of this Jacobian is −ω. The other eigenvalues are the solutions of the characteristic equation of the following submatrix:

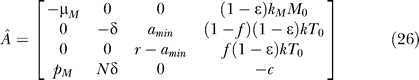
As explained in the previous section, bifurcation conditions can be obtained by finding conditions to make the linear system *ÂX* = 0 singular, where *X* = [M*, T*, *L*, *V*]*^T^*. By Gaussian elimination, this linear system can be reduced to the following equivalent system:

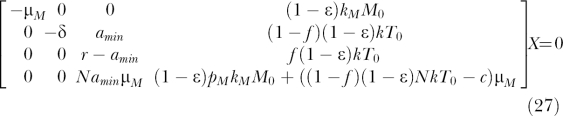
If *r* = *a_min_*, then only ɛ = 1 can make the system singular. If *r* ≠ *a_min_*, then one more step of elementary row operation yields *r*((1 − *f*)(1 − ɛ)*NkT*_0_ − *c* + (1 − ɛ)*p_M_k_M_M*_0_/μ*_M_*) − *a_min_*((1 − ɛ)*NkT*_0_ − *c* + (1 − ɛ)*p_M_k_M_M*_0_/μ*_M_*) as the pivot in this elimination step, which should be equal to zero for this system to be singular. Applying the relation *p_M_k_M_*/μ*_M_* = φ_1_ *c*/*M_ps_*, obtained from the pretreatment quasi–steady state condition dM*/*dt* = *k_M_MV* − μ_M_M* = 0 and φ_1_ = p_M_M_ps_*/*cV_ps_*, gives the following bifurcation condition:

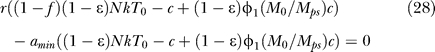
which is valid for all the ɛ, *a_min_*, and ω values. Note that the bifurcation condition is simplified to *r* = 0 or *c* = (1 − *f*)(1 − ɛ)*NkT*_0_ + (1 − ɛ)φ_1_(*M*_0_/*M_ps_*)*c* when *a_min_* = 0, and *r* = *a_min_* when ɛ = 1. Finally, from Equation 28, the critical drug efficacy (ɛ*_c_*) becomes

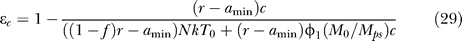
If we apply the pretreatment steady state condition *NkT_ps_* = (1 − φ_1_)*c*, with the assumption that (1 − *f*)*r* − *a_min_* ≈ *r* − *a_min_* when *f* = 3 × 10^−6^, the critical efficacy is simplified to

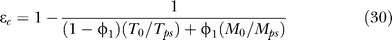
Note that the inclusion of long-lived cells in the model increases the critical efficacy. Intuitively, this makes sense as therapy now needs to block infection in this additional cellular compartment.

### System model and parameters for Figure 6.

We applied Equations 24 in the previous section with *T*_0_ replaced by *T_ps_* to investigate the decay dynamics of HIV-1 from the initiation of ART. Unless otherwise specified, the same pretreatment conditions and parameters given in the main text were applied: *T_ps_* = 486 cells/μl, δ = 1 d^−1^, *c* = 23 d^−1^, *N* = 2 × 10^4^ HIV-1 RNA/cell, *f* = 3 × 10^−6^, φ_1_ = 0.05, φ_2_ = 0.008 (< 0.01 [6]), *r* = −0.00171 d^−1^, ω = 0.00939 d^−1^, *a_min_* = 0, and ɛ = 0.7 (default). For replication-competent latently infected cells and viral load immediately before the initiation of therapy, *L_ps_* = 6 × 10^5^ cells [30,47] and *V_ps_* = 10^5^ HIV-1 RNA copies/ml were applied, respectively. The death rate of productively infected long-lived cells (μ*_M_*) was set to μ*_M_* = 0.07 d^−1^ [6]. Other pretreatment conditions and parameters of the model, such as *a_ps_*, M_ps_*, T_ps_*, *M_ps_*, *k*, *k_M_*, and *p_M_*, were chosen to satisfy the pretreatment quasi–steady state condition. For example, the relation T_ps_* = (1 − φ_1_)*cV_ps_*/(*N*δ) can be obtained from *dV*/*dt* = *N*δT* + p_M_M* − *cV* = 0 and φ_1_ = p_M_M_ps_*/*cV_ps_*, and *a_ps_* = φ_2_δT_ps_*/*L_ps_* from φ_2_ = *a_ps_L_ps_*/δT_ps_*, respectively. Next, we assumed that a constant fraction of viral infection events results in chronic infection, i.e., *k_M_M_ps_* = *αkT_ps_*, where *α* = 0.2 [31] was applied as its baseline value. Then *k_M_M_ps_* can be calculated using *k* = (1 − φ_1_)(1 − φ_2_)*c*/(1 − *f*)*NT_ps_* from Equation 8. Finally, M_ps_* = *k_M_M_ps_V_ps_*/μ*_M_* can be obtained from dM*/*dt* = *k_M_MV* − μ_M_M* = 0, and then *p_M_* is *p_M_* = φ_1_ *cV_ps_*/M_ps_*.

## Abbreviations

ART: antiretroviral therapy
PI: protease inhibitors
RT: reverse transcriptase
δ: death rate of productively infected CD4^+^ T cells
ω: deceleration rate in the activation of latently infected cells
η: deceleration rate in the T cell recovery
λ: replenishment rate of target cells from their source
ɛ: total combined drug efficacy, where (1 − ɛ) = (1 − ɛ*_RT_*)(1 − ɛ*_PI_*)
φ_*1*_: fraction of plasma virus produced by long-lived infected cells at the pretreatment quasi–steady state
φ_*2*_: contribution of latently infected cells to the pretreatment quasi–steady state pool of productively infected CD4^+^ T cells
δ*_L_*: death rate of latently infected cells
μ*_M_*: death rate of productively infected long-lived cells
ɛ*_PI_*: drug efficacy of *PI*s, where 0 ≤ ɛ*_PI_* ≤ 1
ɛ*_RT_,*: drug efficacy of RT inhibitors, where 0 ≤ ɛ*_RT_* ≤ 1
*a*: activation rate of latently infected cells
*a**_min_*: minimum activation rate of latently infected cells
*c*: virion clearance rate from the plasma
*d*: death rate of CD4^+^ T cells
*d_M_*: death rate of long-lived cells
*f*: fraction of new viral infections resulting in latency
*k*: viral infectivity to CD4^+^ T cells
*k_M_*: viral infectivity to long-lived cells
*L*: latently infected cells
*M**: productively infected long-lived cells
*M*: long-lived cells
*N*: number of virions produced during the average lifespan of *T** (in vivo burst size)
*p*: maximum proliferation rate of CD4^+^ T cells in their density-dependent logistic proliferation
*p_bs_*: bystander proliferation rate of latently infected memory CD4^+^ T cells
*p_M_*: virus production rate from productively infected long-lived cells
*ps*: a subscript denoting a pretreatment quasi–steady state value
*r*: net regeneration rate of latently infected cells, where *r* = *p_bs_* − δ*_L_*
*s_M_*: production rate of long-lived cells from their source
*T**: productively infected CD4^+^ T cells
*T*: CD4^+^ T cells that are susceptible to infection (target cells)
*T_0_*: T cell concentration at the beginning of simulation (3 mo after initiation of ART)
*T_max_*: T cell density at which its proliferation shuts off
*T_ps_*: pretreatment T cell concentration
*T_sat_*: T cell concentration that *T* approaches as t → ∞ under suppressive ART
*V*: total plasma viral load, where *V* = *V_I_* + *V_NI_*
*VI*: infectious virus
*V_NI_*: noninfectious virus produced by the action of PIs
